# Independent and combined effects of fine particulate matter and greenness on autism spectrum disorder symptoms: investigating sensitive periods of exposure in the early two years of life

**DOI:** 10.3389/fped.2025.1561476

**Published:** 2025-04-10

**Authors:** Yi Liu, Wensu Zhou, Meng Liu, Yichao Wang, Shu Chen, Xiyue Xiong

**Affiliations:** ^1^ NHC Key Laboratory of Birth Defect for Research and Prevention, Hunan Provincial Maternal and Child Health Care Hospital, Changsha, China; ^2^ School of Public Health, Sun Yat-sen University, Guangzhou, China; ^3^ School of Xiangya Public Health, Central South University, Changsha, China

**Keywords:** air pollution, greenness, autism spectrum disorder, symptoms, child health

## Abstract

**Background:**

The impact of exposure to fine particulate matter (aerodynamic diameter ≤2.5 μm, PM_2.5_) and greenness during early two year of life on Autism Spectrum Disorder (ASD) symptoms, especially under the combined influence of the two factors, and the sensitive periods of exposure during the early life, remain underexplored.

**Objective:**

This cross-sectional study recruited 108 children with ASD and aimed to quantify the independent and combined effects of PM_2.5_ and greenness exposure on ASD symptoms during the first two years of life.

**Methods:**

We collected PM_2.5_ levels and Normalized Difference Vegetation Index (NDVI) values to reflect PM_2.5_ exposure and greenness levels, meanwhile, assessing ASD symptoms with the Autism Behavior Checklist (ABC) and its sub-scales (sensory, relating, stereotypic behavior, language, and social independence) scores. We identified six sensitive exposure periods: 6 months, 7–12 months, 13–18 months, 19–24 months after birth, and the first and second years after birth. We investigated the independent effects of PM_2.5_ and greenness on ASD symptoms using multiple linear or logistic regression for continuous or categorical symptom scores, and explored their additive interaction and mediation effects.

**Results:**

Multiple linear models showed reduced total ABC, relating, and social independence scores with greenness exposure at 19–24 months after birth, while 7–12 months and first year exposures benefited social independence. Logistic models showed that PM_2.5_ exposures during 13–18 months after birth increased symptoms of stereotypic behavior, while low greenness exposure during 19–24 months after birth heightened the risk of social independence impairment. We found high levels of PM_2.5_ and low greenness during the 13–18 months after birth increased the risk of overall severity. Greenness exposure during 6 months after birth could mitigate the effects of PM_2.5_ exposures during 13–18 months.

**Conclusion:**

Our findings underscore the importance of reducing air pollution and enhancing greenness to mitigate ASD symptoms.

## 1 Introduction

Autism Spectrum Disorder (ASD) is characterized by early-onset difficulties in social communication and a distinctive pattern of restricted, repetitive behaviors and interests (1). It is a complex neural disorder, and recent evidence suggests that approximately 1 in 100 children globally are diagnosed with ASD (2). The multifaceted etiology of ASD underscores the importance of addressing the adverse impacts of contributing factors. Previous studies have found that air pollution, which consists of a mixture of several components including gases, organic compounds, and ambient particulate matter (PM), significantly affects the development of ASD (3). Of particular concern is fine particulate matter (PM_2.5_), which exerts the most substantial impact. Recent studies utilizing case-control and birth cohort designs have demonstrated a significant association between prenatal and postnatal PM_2.5_ exposure and elevated autism spectrum disorder (ASD) risk (3–7). Potential mechanisms linking air pollutants and ASD including the detrimental impact of particulate matter on the immune system leading to neuroinflammatory responses and oxidative stress, which are associated with ASD-like behaviors (8); complex gene-environment interactions and epigenetic factors (9, 10); and other potential molecular mechanisms like abnormalities in oxidative stress biomarkers, such as catalase (CAT) (11).

In addition to the risk of ASD, there is evidence indicating a connection between PM_2.5_ and the exacerbation of ASD symptoms (12, 13). This is significant because the considerable clinical diversity observed in individuals with autism highlights the need to understand how environmental factors impact the condition (3), although this area of research is not as thoroughly explored. Moreover, the early-life exposure to air pollution is particularly concerning, considering the critical development phase of the brain post-birth. Indeed, ASD typically manifests within the first two years of life and previous studies have shown strong evidence of a heightened risk for ASD associated with exposure to PM_2.5_ during this period (6, 10). However, findings regarding the impact of PM_2.5_ exposure during first two years of life on ASD symptoms have been inconsistent across studies (12–14). When examining the impact of air pollutants on ASD risk or symptom severity, the exposure window is often direct defined as the first or two years after birth (12, 13, 15–17). Nevertheless, a conceivable scenario is that individuals who developed ASD prior to the age of diagnosis are not influenced by the air pollution they were exposed to after this age. It is necessary to accurately assess the exposure level by taking into account the age at which the diagnosis was made. Understanding the temporal aspects of these associations is vital for identifying critical exposure periods and for avoiding measurement bias in research. Furthermore, it is also intriguing to explore the impact of air pollution exposure prior to diagnosis on the severity of ASD. It can offer evidence of the long-term, short-term, or incubation effects of air pollution. However, current discussions on the relationship between pre-diagnosis exposure of PM_2.5_ and ASD symptoms are inadequate (6).

Previous studies have indicated that exposure to green spaces is beneficial for early childhood development, cognitive growth, physical and mental health, this topic has become particularly significant against the background of their continual degradation caused by urbanization (18–20). Indeed, green spaces offer a range of health benefits and promote well-being through both direct and indirect pathways. They facilitate physical activity and social interaction, and help mitigate environmental factors like noise, heat, and air pollution (21, 22). In relation to enhancing neurodevelopment in autistic children, interaction with natural environments serves as a restorative factor that stimulates essential emotional states and improves gut health, which closely related to the reduction of ASD symptoms (23–25). Epidemiological evidence supports that access to green spaces may decrease the likelihood of ASD, yet, the impact of green spaces on the severity of ASD remains under-researched (18, 26, 27).

Although to date, several studies have examined the potential of greenness to mediate the effects of air pollution on ASD prevalence (26, 28), the potential interactive and mediating effects of greenness on the relationship between PM_2.5_ and ASD symptoms also warrant quantification. Let alone, PM_2.5_ also can mediate the effect of greenness on health outcomes (29, 30), but relevant evidence on the symptoms of ASD is limited. An additional area of interest is the role of green spaces throughout the early life of children with ASD, specifically whether exposure to green spaces can counteract the harmful effects of concurrent air pollution exposure or offer enduring protection against the detrimental impacts of subsequent harmful exposures, thereby potentially leading to a reduction in autistic symptoms. This hypothesis gains plausibility from the enduring benefits of green spaces on child development, which begin in early life and persist into older age (19, 31).

Therefore, by analyzing effects of multiple exposure windows, our formalized hypothesis is that exposure to PM_2.5_ increases the risk of more severe symptoms of ASD. Additionally, greater greenness can reduce the symptoms of ASD. Furthermore, there are combined effects of PM_2.5_ and greenness on symptoms, including interaction and mediation effects.

## 2 Materials and methods

### 2.1 Study design, participants and produces

Our cross-sectional study is a part of the China Multi-Center Children Autism Project (CMCAP), with the specific dataset employed in our research being sourced from Hunan Province; detailed description has been published elsewhere (32). We recruited participants from Changsha city, the capital of Hunan, during the period from March 2018 to May 2019. Changsha is a major megacity in Central China with a high level of air pollution and rapid urbanization (urbanization rate was 84% in 2022). Through random sampling design, stratified by district, we selected four special education institutions from a total of ten for our research. These institutions, located across four of six main urban districts of Changsha (Tianxin, Kaifu, Wangcheng, Yuhua), are recognized for their relative substantial size and function as designated centers for disabled children's rehabilitation and rescue assistance. These institutions were identified through the government's department (Hunan Disabled Persons' Federation). These centers are adept at providing behavioral and speech therapy to autistic children under 12 years old. All ASD children were recruited from special education institutions but primarily resided at home, with their family serving as their main caregivers.

The participants for this study were selected from children with a diagnosis of ASD documented at these centers. This diagnosis was verified through outpatient or inpatient records provided by a psychiatrist, psychologist, or neurodevelopmental pediatrician from a tertiary hospital. The inclusion criteria specified children formally diagnosed with ASD according to the DSM-V or DSM-IV criteria, excluding Pervasive Developmental Disorder-Not Otherwise Specified (PDD-NOS) and Asperger Syndrome. Additionally, eligible participants were those older than 24 months at the time of the interview (as early ASD symptoms are typically identified between 12 and 24 months), who had resided in the survey city from birth until the interview (ensuring a consistent and long-term exposure assessment), and who consented to participate in the study. Ultimately, 108 children with ASD, including 24 females and 84 males were enrolled in the study. The participants reside across all six major districts of the city. All provided their residence of home addresses, which at district-level and geocoded with a 6-digit zip code. Hence, this study assumed that subjects residing in the same district experienced same levels of exposure.

We collected demographic characteristics of children using a structured questionnaire. The primary caregivers of the children, such as parents or grandparents, provided their informed consent after receiving a comprehensive explanation of the study's purpose. Trained graduate students from the Xiangya School of Public Health at Central South University assisted the participants in completing the questionnaire. This assistance included clarifying questions and helping to record information. The interviewee further provided detailed information about the age at clinical diagnosis of ASD based on the hospital records. In this paper, we consider the diagnosis age of ASD made by medical institutions as the incidence time of ASD. Because we believe that this indicator offers a relatively accurate assessment for ASD, distinguishing it from developmental delays and other neurological disorders that may complicate the diagnosis of ASD. Additionally, in such a city survey site, the majority of parents possessed at least a high school education and would seek medical assistance upon noticing early symptoms. In our sample, over half of the subjects (75%) were diagnosed with ASD before the age of 3, which is close to the onset of early ASD symptoms. Ethics approval was provided by the Ethics Committee of Hunan Provincial Maternal and Child Health Care Hospital (EC20180318).

### 2.2 ASD symptoms

The evaluation of ASD symptoms was conducted using a widely recognized tool, the Autism Behavior Checklist (ABC). This instrument is frequently used to assess the core symptoms and severity of ASD. Demonstrating reliable and valid results, the ABC is suitable for children aged over 18 months and enables the assessment of symptoms in autistic children, with their caregivers completing the checklist. It comprises a 57-item checklist, categorized into five dimensions: sensory, relating, stereotypic behaviour, language, and social independence. Each item is rated on a scale from 1 to 4, reflecting the severity of symptoms. The screening threshold is set at a score of 53, with scores of 68 or higher indicating a likely ASD diagnosis (33). Higher scores are associated with more severe challenges. According to the Autism Diagnosis, Treatment, and Rehabilitation guidelines in China, a score below 31 is deemed normal. This benchmark was established based on studies with Chinese children, where a cut-off of 31 maintained the scale's reliability and validity (34, 35). This cut-off was chosen for this study, considering potential biases such as mothers' misinterpretations of items or failure to recognize autistic behaviors in their children. Therefore, in this study, scores calculated from ABC scales were continues variables and scores ranging from 31 to 67 are defined as indicative of mild symptoms, while scores of 68 or above suggest moderate to severe symptoms (36).

### 2.3 Assessment of Pm_2.5_ and greenness exposure

**Figure 1:**
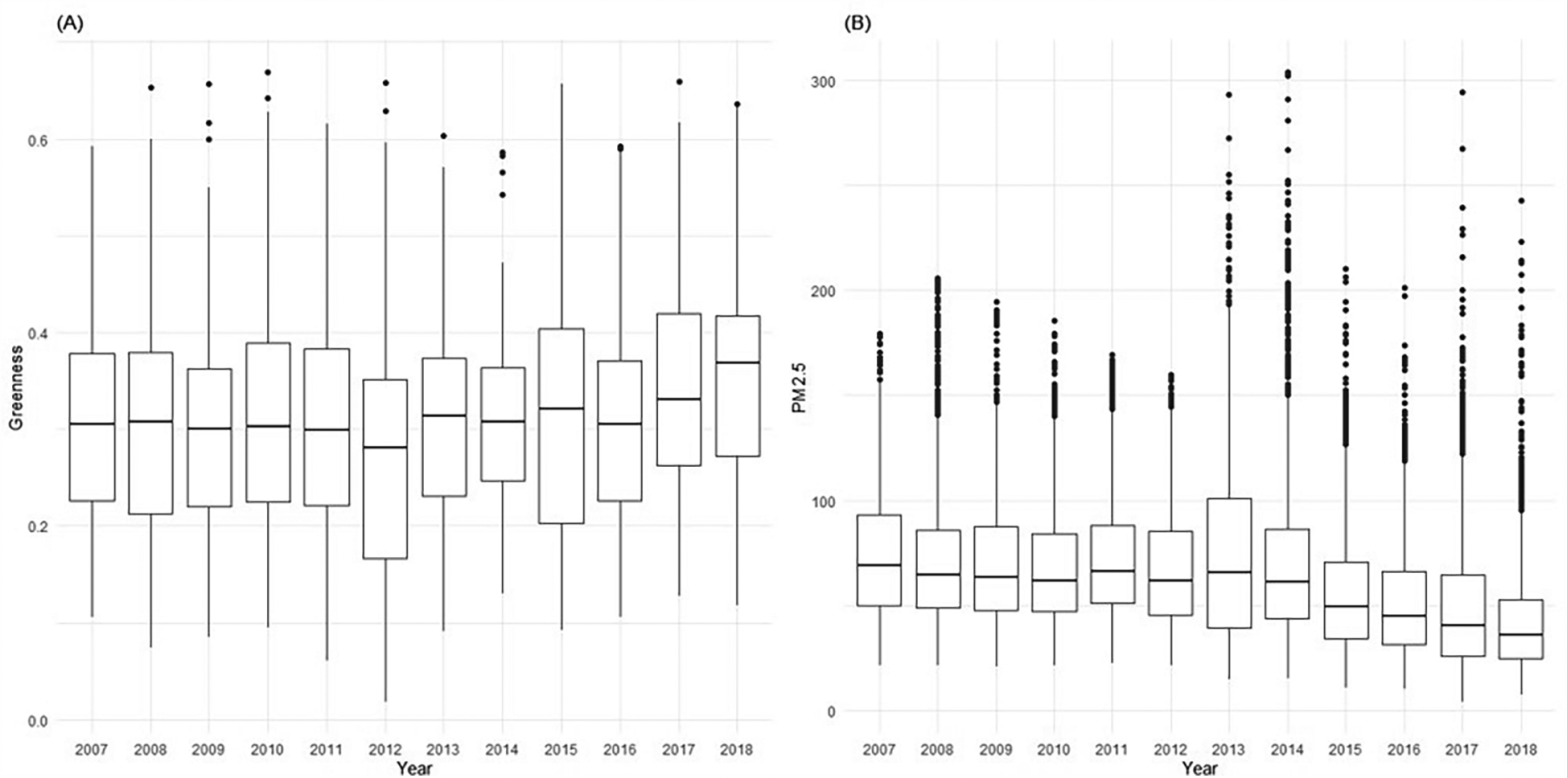
Boxplots of monthly greenness **(A)** and daily PM_2.5_ **(B)** of Changsha city during 2007–2018.

The daily PM_2.5_ and PM_10_ concentration data from 2007 to 2018 were obtained from the CHAP (China High Air Pollutants) database (37, 38), which provided mean daily estimates of ambient PM_2.5_. The PM_2.5_ data is part of the China High Air Pollutants series, a comprehensive dataset of ground-level air pollutants in China known for its long-term coverage, high resolution, and quality. It boasts a high accuracy level, evidenced by a cross-validation coefficient of determination (CV-R^2^) of 0.92, a root-mean-square error (RMSE) of 10.76 µg/m^3^, and a mean absolute error (MAE) of 6.32 µg/m^3^ on a daily basis (37). Our research primarily focused on PM_2.5_, hence, PM_10_ data was incorporated in a sensitivity analysis to validate the impact of particle size. For evaluating exposure to greenery, we utilized the Normalized Difference Vegetation Index (NDVI), a well-established measure for assessing vegetation coverage. NDVI reflects the extent of greenness, considering the quantity and spatial distribution of trees and vegetation. It is calculated using land surface reflectance in the visible (red) and near-infrared parts of the spectrum: NDVI = (NIR − red)/(NIR + red). We acquired NDVI data from NASA's Earth Observing System's global Moderate Resolution Imaging Spectroradiometer (MODIS) via Google Earth Engine, which offers a spatial resolution of 500 m × 500 m (MOD13A1). The NDVI values, ranging from −1 to 1, with higher positive values indicating denser vegetation. For our study, we used monthly NDVI values to assess exposure, reflecting the real-world rate of vegetation growth. Figure 1 presents monthly greenness and daily PM_2.5_ of Changsha city during 2007–2018.

### 2.4 Exploration on the sensitive exposure window

Our study primarily focuses on the exposure periods during the first two years of life. Thus, we segmented the exposure period of participants into six stages based on their age at ASD diagnosis and birth dates, calculating mean exposures for these intervals. According to the previous study (6), the exposure windows were defined as follow: 6 months after date of birth (Stage 1), 7 months after date of birth - 12 months after date of birth (stage 2), 13 months after date of birth - 18 months after date of birth (stage 3), 19 months after date of birth - 24 months after date of birth (stage 4), first year after birth (i.e., age 1) (stage 5), second year after birth (i.e., age 2) (stage 6). Notably, the sample sizes varied across these exposure windows due to differences in age at diagnosis. For example, participants diagnosed with ASD after 12 months had exposure experiences in Stages 1 and 2 (*n* = 108), but unaffected by exposure in Stage 3. These details are listed in Table 1, indicating that various sub-samples were identified for separate analyses. Additionally, we did not consider exposures occurring before the diagnosis as they showed less or no significant association with ASD symptoms, as indicated in Supplementary Table S1.

**Table 1:** Summary of the sensitive windows of exposure.

Time period	Exposure window period	*N*	PM_2.5_, median (IQR)	Greenness, median (IQR)
Stage 1	6 months after date of birth	108	65.7 (58.9, 78.6)	0.28 (0.24, 0.38)
Stage 2	7 months after date of birth - 12 months after date of birth	108	55.6 (43.5, 65.5)	0.33 (0.27, 0.39)
Stage 3	13 months after date of birth - 18 months after date of birth	103	61.0 (48.3, 67.2)	0.28 (0.25, 0.37)
Stage 4	19 months after date of birth - 24 months after date of birth	89	47.7 (39.6, 63.5)	0.32 (0.26, 0.39)
Stage 5	First year after birth (i.e., age 1)	108	62.3 (63.2, 70.2)	0.29 (0.27, 0.36)
Stage 6	Second year after birth (i.e., age 2)	108	52.1 (49.3, 63.4)	0.30 (0.26, 0.38)

### 2.5 Covariates

In our study, we identified relevant covariates associated with ASD risk based on prior research (12, 15, 39). These covariates include the child's sex (boy or girl), race/ethnicity (Han or minority), maternal education level (junior high school or less, high school, college graduate or higher), maternal age at delivery (≤27, 28–30, >30), age of male parents (≤27, 28–30, >30), maternal occupation (employed or unemployed), maternal marital status (married or unmarried), birth year (categorized into four periods: 2007–2011, 2012–2013, 2014, 2015, and 2016), the season of birth (hot months, from May to September) or cold months. We included birth year as an important factor because previous study has reported that the risk of ASD is highest for fall births (i.e., conceived in the winter) and lowest for spring births (i.e., conceived in the summer) (40). Based on the findings of a published survey, we chose not to include gestational week (<37, 37–41, >41) as a confounding factor in our model. This decision was made on the grounds that gestational week might be part of the causal pathway linking air pollution to ASD (39).

### 2.6 Statistical analyses

We described the demographic characteristics of the children in our study. Categorical variables were presented as numbers and percentages. For continuous variables, we reported the mean ± standard deviation (SD) when they conformed to a normal distribution. Alternatively, if the distribution was not normal, we calculated the median ± interquartile range (IQR).

Our analysis focused on examining the association between PM_2.5_, greenness, and ASD symptoms, measured by the total scores of the ABC and its subscale scores for specific symptoms. These scores were log-transformed to address skewness and to meet the assumptions of multiple linear regression models, as done in previous study (13). We reported the results for each 10-unit increase in PM_2.5_ and per 0.1-unit increase in NDVI. Separate models were executed for each sensitive window period and we reported the *β* with a 95% confidence interval (95% CI) for effect estimations. We computed variance inflation factors (VIFs) to quantify multicollinearity between the exposures and covariates, and we did not find any violation of assumptions in the analysis. Using total scores as an example, the VIFs range from 1.03 to 1.11, which showed not multicollinearity existed. To further elucidate the findings, we examined potential dose-response relationships using restricted cubic spline (RCS) models with 3 knots.

Next, we referred to the 68 cut-off of the ABC to categorize severity levels as binary variables, distinguishing between milder and moderate to severe severity. Each specific symptoms of the ABC was similarly divided into two groups based on its median value as: higher severity (≥Median) and milder severity (<Median). To analyze the impact of PM_2.5_ and greenness on these binary symptom variables, we employed logistic regression models. This approach allows for a nuanced understanding of how these environmental factors might affect the likelihood of more severe ASD symptoms as categorized by the ABC scores. These models were utilized to calculate the odds ratios (OR) and 95%CI for effect estimation. Given the nested structure of our data, with individual participants situated within various districts, we initially contemplated incorporating multilevel regression into our analysis. We started with a “null” model analysis, which employed either continuous scores or binary variables of the ABC as the dependent variable without including any independent variables. The null model revealed Intra-class Correlation Coefficients (ICCs) of 0.004 for ABC total scores and less than 0.001 for categorical variables, hence, little of the variation in symptoms were attributable to differences at the district level. This led us to conclude that the use of multilevel models was not warranted for further analysis.

In our study, we conducted interaction analyses to investigate the combined effects of PM_2.5_ and greenness on the severity of ASD. We primarily focused on examining the additive interactions between PM_2.5_ and greenness on ASD symptoms. Because is the more relevant public health implication and the estimated interaction on an additive scale better reflect biological interaction (41). The dependent variables in this model were the binary variables of the overall and specific scores of the ABC, as previously described. Herein, PM_2.5_ and NDVI levels were transformed into categorical variables based on their median values: high level (≥Median) and low level (<Median). To evaluate the potential interaction, we calculated the relative excess risk due to interaction (RERI) using the formula: RERI = OR_11_ − OR_10_ − OR_01_ + 1. Here, OR_11_ represents the combined exposure to high levels of PM_2.5_ and low levels of NDVI, while OR_01_ and OR_10_ denote the presence (1) or absence (0) of each exposure separately. A positive RERI value (RERI > 0) suggests a synergistic effect, indicating a positively additive interaction. Conversely, a negative RERI value (RERI < 0) indicates an antagonistic and negatively additive interaction. If the 95% CI for RERI includes 0, it suggests null additive interaction.

Finally, we employed mediation analyses to investigate the potential mediating role of greenness/PM_2.5_ in the relationship between PM_2.5_/greenness and the overall and specific symptoms using causal mediation analyses. In this model, PM_2.5_/greenness served as the independent variable (X), and greenness/PM_2.5_ was considered the mediator (M). Total effects are categorized into average causal mediation effects (ACME) and average direct effects (ADE), namely, two components including the indirect effect (through M) and the direct effect (effect of X on ASD severity not mediated by M). The indirect effects evaluate the relationship between PM_2.5_/greenness and the overall and specific symptoms, mediated by greenness/PM_2.5_. A nonparametric bootstrap method with 1,000 resamples was employed to test the mediation estimates. Considering the numerous combinations of X and M across various exposure windows, our mediation analyses were grounded in the significant findings of the primary analysis. Specifically, we concentrated on the significant associations between PM_2.5_/greenness and symptoms as our total effects.

Lastly, to ensure the robustness of our findings, we adopted several strategies for validation. We used PM_10_ as an alternative proxy for air pollution to confirm the effects observed with PM_2.5_ on ASD symptoms. Additionally, we repeated significant analyses by altering the cut-off for the total ABC scores (using a threshold of <53 vs. ≥53) to assess the consistency of our results across different severity categorizations. All statistical analyses were conducted using R (version 4.2.2; R Development Core Team). We considered a two-sided *p*-value of less than 0.05 as indicative of statistical significance.

## 3 Result

### 3.1 Characteristic of participants

Table 2 describes the general characteristics of the participants in our study. The study consisted of 108 children with a mean age of 4.57 years (SD = 1.76) at the time of the interview. The sample included more boys (77.8%) than girls. The majority of mothers were aged over 30 years at the time of delivery and had attained a high school education or higher education level. The mean scores of the ABC were recorded as 61.00 (IQR = 31.25). As exposure in stage 1 an example (see Table 1), the median concentrations of PM_2.5_ and NDVI were 65.7 μg/m^3^ (IQR = 19.7) and 0.28 (IQR = 0.14), respectively.

**Table 2:** Distribution of children's general information and ASD symptoms.

Variables	Categories	*n* (%)
Gender	Boy	84 (77.8)
	Girl	24 (22.2)
Age at interview (year)	4.57 ± 1.76	
	<4	29 (26.9)
	4–6	64 (59.3)
	>6	15 (13.9)
Race/ethnicity	Han	96 (88.9)
	Minority	12 (11.1)
Education level	Junior high school or less	12 (11.1)
	High school	42 (38.9)
	University degree or above	54 (50.0)
Occupation	Employed	62 (57.4)
	Unemployed	46 (42.6)
Age at delivery (year)	<27	29 (26.9)
	27–30	37 (34.3)
	>30	42 (38.9)
Age of male parents (year)	<27	11 (10.2)
	27–30	64 (59.3)
	>30	33 (30.6)
Year of birth	2007–2011	14 (13.0)
	2012–2013	27 (25.0)
	2014	32 (29.6)
	2015	22 (20.4)
	2016	13 (12.0)
Season of birth	Hot months	47 (43.5)
	Cold months	61 (56.5)
Gestational week (week)	<37	14 (13.0)
	37–41	87 (80.6)
	>41	7 (6.5)
Marital status	Married	103 (95.4)
	Others	5 (4.6)
Parity	1	36 (33.3)
	>2	72 (66.7)
Autism behavior checklist (ABC)	ABC total score	61.00 (45.00, 76.25)
	Sensory score	8.00 (4.00, 12.00)
	Relating score	13.00 (8.00, 19.00)
	Stereotypic behaviour score	11.00 (5.00, 17.00)
	Language score	16.50 (8.00, 26.00)
	Social independence score	11.00 (8.75, 16.00)
ASD severity	Milder severity	67 (62.0)
	Moderate to severe severity	41 (38.0)

### 3.2 Associations of Pm_2.5_ and greenness with ASD severity

**Figure 2:**
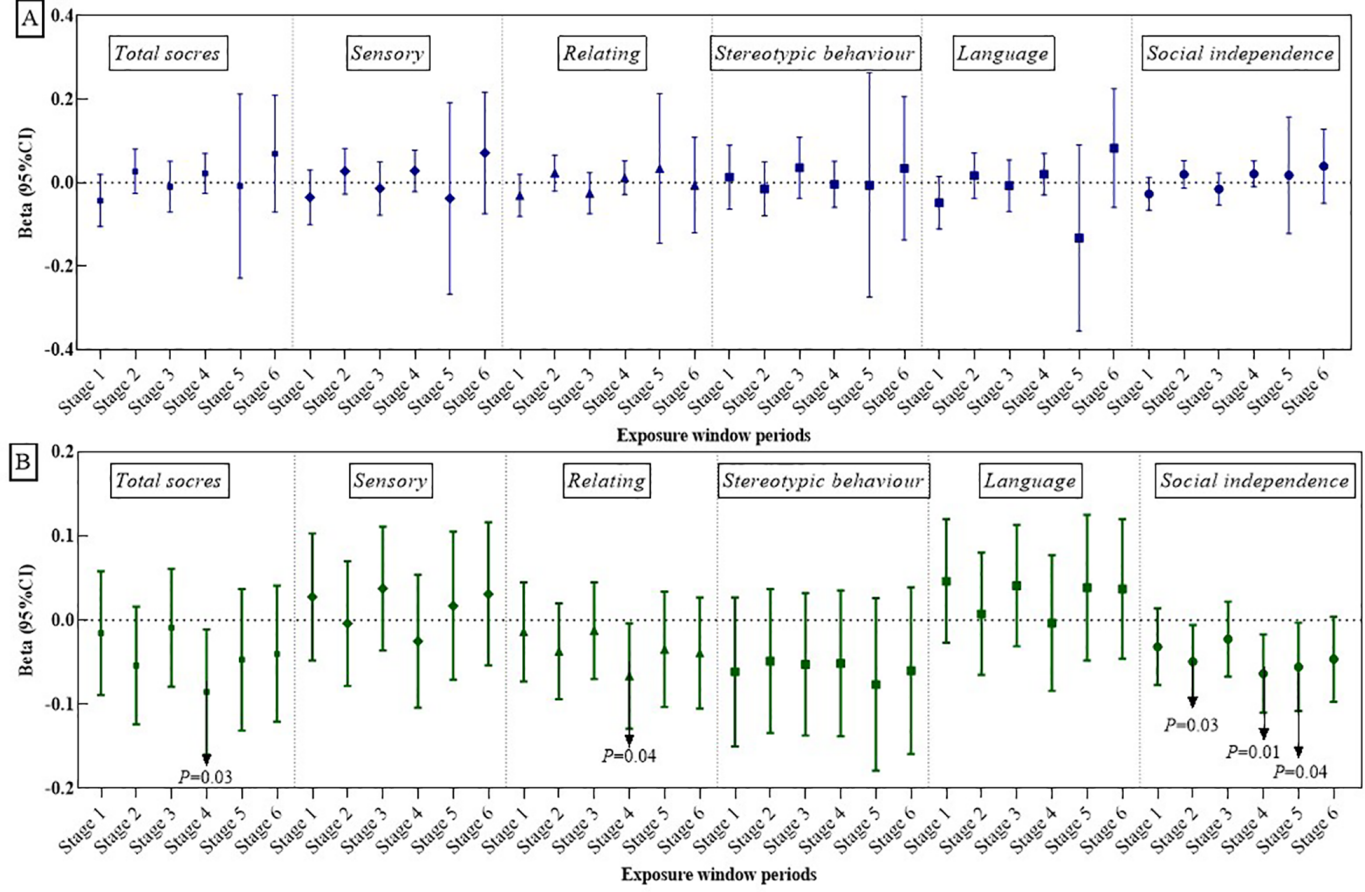
The relationships of PM_2.5_ exposure **(A)** and greenness exposure **(B)** with total ABC scores and its specific sub-scores.

**Figure 3:**
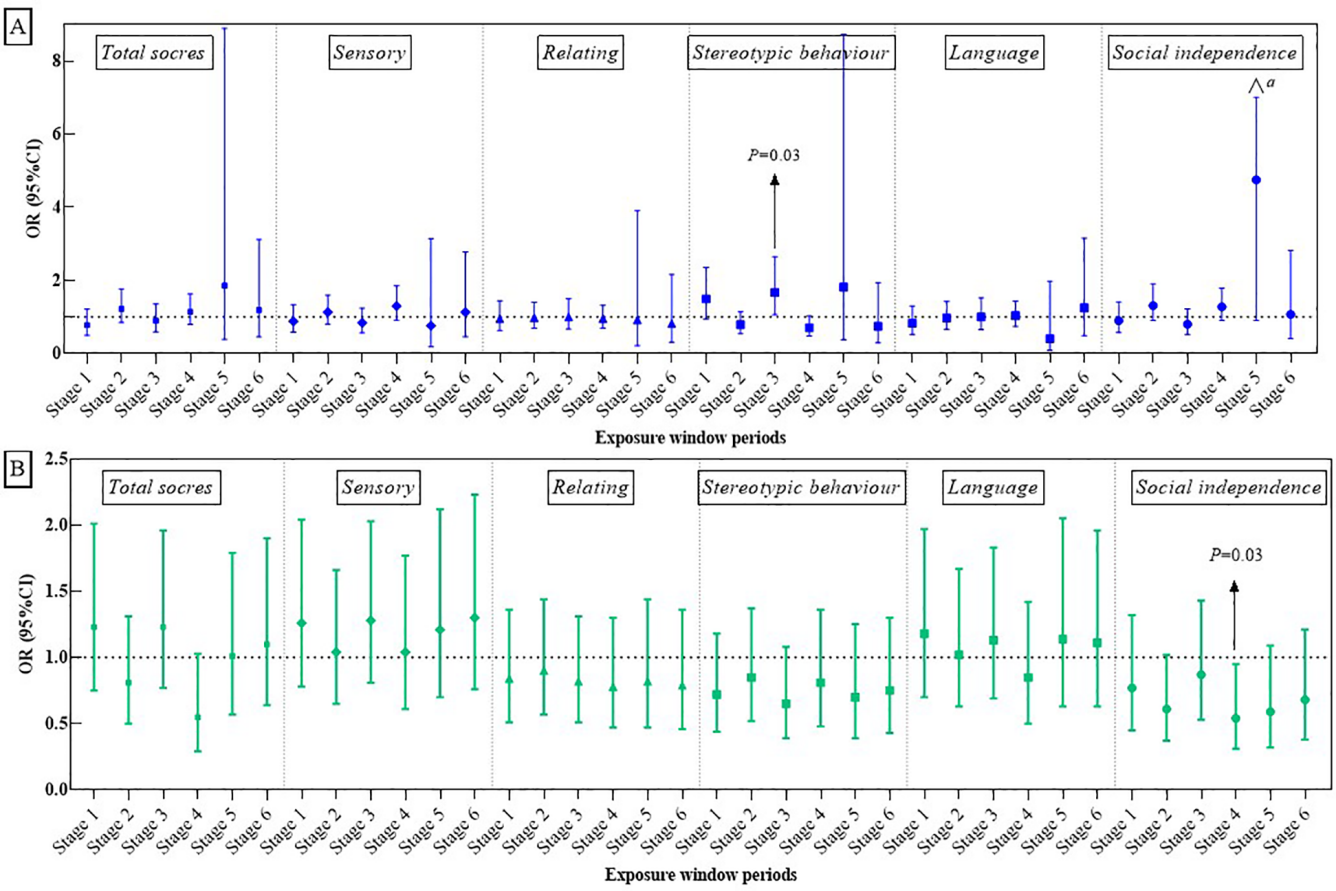
Associations of PM_2.5_ **(A)** and greenness exposure **(B)** with overall and domain-specific ASD symptom categories (categorical variables). a = 24.98.

Figures 2A, 3A illustrate the association between PM_2.5_ exposures and ASD symptoms by linear and logistic regression models, respectively. No significant relationship was found between PM_2.5_ exposures and the total scores or specific scores (Figure 2A). However, a positive association was observed when considering the stereotypic behavior scores as a binary variable. To be specific, exposure to PM_2.5_ between 13 and 18 months after birth (Stage 3) was linked to an increased likelihood of more severe stereotypic behaviour symptoms (OR: 1.67, 95% CI: 1.06–2.64, *P* = 0.03, see Figure 3A).

Figures 2B, 3B present the impact of greenness on ASD severity. Exposure to greenness between 19 and 24 months after birth (Stage 4) was significantly associated with a lower risk of more severe symptoms (*β*: −0.09, 95% CI: −0.16, −0.01, *P* = 0.03 for total scores; *β*: −0.07, 95% CI: −0.13, −0.00, *P* = 0.04 for relating; *β*: −0.07, 95% CI: −0.11, −0.02, *P* = 0.01 for social independence). Notably, each 0.1-unit increase in greenness during this period was significantly associated with a decreased occurrence of moderate and higher severity symptoms in the social independence scale (OR: 0.54, 95%CI: 0.31, 0.95, *P* = 0.03). Linear models (Figures 2B) further showed reduced social independence scores with greenness exposure at 7–12 months after birth (Stage 2) (*β*: −0.05, 95% CI: −0.09, −0.01, *P* = 0.03) and exposure at age 1 (Stage 5) (*β*: −0.06, 95% CI: −0.11, −0.02, *P* = 0.04) benefited social independence. According to the dose-response relationship analysis, we did not find strong evidence of non-linear associations between PM_2.5_ and greenness with ASD symptoms (Supplementary Table S2).

### 3.3 Interaction effects of Pm_2.5_ and greenness on ASD severity

Based on logistic regression models, we examined the additive interaction effects between PM_2.5_ and NDVI during sensitive window periods on ASD symptoms (Supplementary Table S3–S4). Herein, we documented significant additive interactions in Table 3. We observed a synergistic effect of high PM_2.5_ during 13–18 months after birth (Stage 3) and low greenness during 6 months after birth (Stage 1) on ASD symptoms (RERI: 1.75, 95% CI: 0.33, 3.17). For high PM_2.5_ and low greenness during Stage 3, the estimates suggested a synergistic effect, suggesting that joint effects were more than expected for additive risks (RERI: 1.44, 95% CI: 0.42, 2.45). Additionally, significant synergistic effects were found for high levels of PM_2.5_ during Stage 3 and low levels of greenness after Stage 1 on language scores (RERI: 1.40, 95%CI: 0.09, 2.72).

**Table 3:** Additive interaction between PM_2.5_ and greenness exposure on ASD symptoms based on logistic regression models.

Exposure window period for PM_2.5_	Exposure window period for NDVI	*N*	Estimate (95% CI)		
13 months after date of birth - 18 months after date of birth (Stage 3)	6 months after date of birth (Stage 1)	103			
Low - PM_2.5_ (<P_50_)	High - NDVI (≥P_50_)		1 [Ref]		
Low - PM_2.5_ (<P_50_)	Low - NDVI (<P_50_)		0.23	0.04	1.24
High - PM_2.5_ (≥P_50_)	High - NDVI (≥P_50_)		0.25	0.04	1.23
High - PM_2.5_ (≥P_50_)	Low - NDVI (<P_50_)		1.23	0.34	4.54
RERI for total scores (<68 and ≥68)			1.75	0.33	3.17
13 months after date of birth - 18 months after date of birth (Stage 3)	13 months after date of birth - 18 months after date of birth (Stage 3)	103			
Low - PM_2.5_ (<P_50_)	High - NDVI (≥P_50_)		1 [Ref]		
Low - PM_2.5_ (<P_50_)	Low - NDVI (<P_50_)		0.29	0.05	1.44
High - PM_2.5_ (≥P_50_)	High - NDVI (≥P_50_)		0.21	0.04	0.93
High - PM_2.5_ (≥P_50_)	Low - NDVI (<P_50_)		0.93	0.25	3.42
RERI for total scores (<68 and ≥68)			1.44	0.42	2.45
13 months after date of birth - 18 months after date of birth (Stage 3)	6 months after date of birth (Stage 1)	103			
Low - PM_2.5_ (<P_50_)	High - NDVI (≥P_50_)		1 [Ref]		
Low - PM_2.5_ (<P_50_)	Low - NDVI (<P_50_)		0.37	0.07	1.83
High - PM_2.5_ (≥P_50_)	High - NDVI (≥P_50_)		0.39	0.06	2.19
High - PM_2.5_ (≥P_50_)	Low - NDVI (<P_50_)		1.16	0.32	4.15
RERI for scores of language (<16.5 and ≥16.5)			1.40	0.09	2.72

Note: Characters in bold means statistical significance at *p* = 0.05. PM_2.5_/NDVI was divided into two categories: <Median and ≥median; ABC was divided into two categories: <68 and ≥68; RERI relative excess risk due to interaction. Models were adjusted for gender of children, race/ethnicity, parity, maternal age at delivery, age of male parents, maternal marital state, maternal occupation, maternal education level, year of birth, and season of year.

### 3.4 Mediation effects of Pm_2.5_ and greenness ASD severity

**Figure 4:**
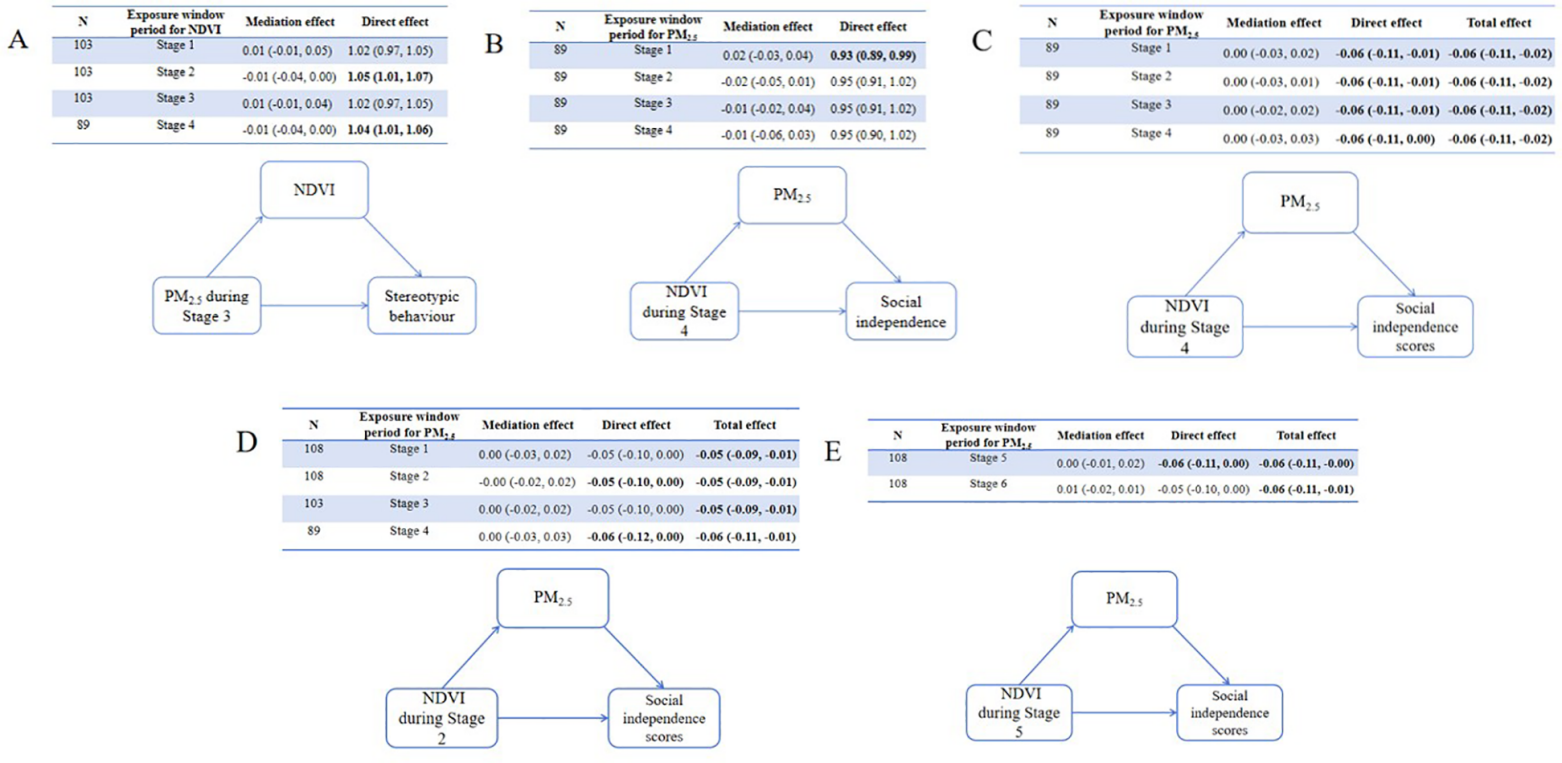
Results of mediation analyses (estimate, 95% CI) for PM_2.5_ on the associations **(A)** between greenness during stage 4 and total ABC scores, **(B)** between greenness during stage 4 and relating scores, **(C)** between greenness during stage 4 and social independence scores, **(D)** between greenness during stage 2 and social independence scores, **(E)** between greenness during stage 5 and social independence scores.

**Figure 5:**
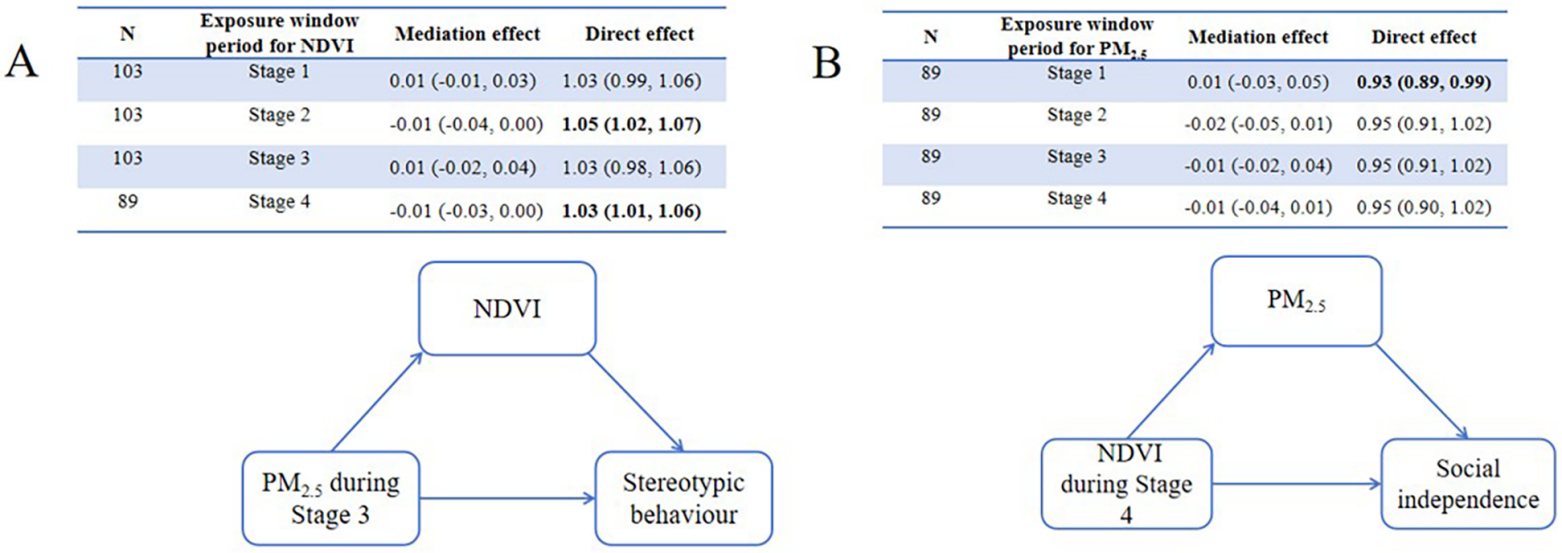
Mediation of associations (estimate, 95% CI) between residential greenness PM_2.5_ and ASD symptoms, by PM_2.5_ greenness. **(A)** Mediation analyses for greenness on the associations between PM_2.5_ during Stage 3 and stereotypic behaviour, **(B)** mediation analyses for PM_2.5_ between greenness during Stage 4 and social independence.

Causal mediation analyses were conducted to explore the role of greenness/PM_2.5_ in the relationship between PM_2.5_/greenness and ASD symptoms (see Figures 4, 5). All analyses were conducted based on the main effects results for the association between PM_2.5_/greenness and ASD symptoms (refer to Figures 2, 3). Figure 4 illustrates the impact of PM_2.5_ on the relationship between greenness and ASD symptoms (measured by continuous scores), where no significant mediation effects were observed (the 95%CI for mediation effects overlap the null hypothesis). Likewise, no significant mediation effects were found in Figure 5.

### 3.5 Sensitivity analyses

In our sensitivity analyses, associations between PM_10_ and stereotypic behavior symptoms were identified (OR: 1.51, 95%CI: 1.04–2.20, *P* = 0.03, see Supplementary Figure S2A), consistent with our main findings. By altering the cut-off for total ABC scores (defined as <53 vs. ≥53), we did not find a significant association between PM_2.5_ exposures, greenness, and ASD symptoms (Supplementary Figure S3), with consistent results observed for the main analysis. Regarding interaction effects, altering the cut-off for total ABC scores did not change our results (Supplementary Table S5). Additionally, the additive interaction of PM_10_ and greenness was weaker than the combined effects of PM_2.5_ and greenness reported in our analysis (Supplementary Table S5). These findings suggest that the sensitivity analyses did not substantially alter our initial findings.

## 4 Discussion

To the best of our knowledge, this study is the first to examine the independent and combined effects of PM_2.5_ and greenness on ASD symptoms, focusing especially on critical exposure periods in the first two years of life. The research reveals three key findings. First, exposure to PM_2.5_ is linked to a heightened risk of more severe ASD symptoms in stereotypic behavior scale. In contrast, greenness seems to reduce the severity of overall ASD symptoms, relating and social independence impairment. Second, the most crucial exposure periods are mainly in the second year of life, with fewer vital periods in the first year. Third, high PM_2.5_ levels combined with low greenness are associated with more severe ASD symptoms. Besides, significant additive effects were observed in various combinations of exposure periods. This study introduces a new perspective and contributes to the existing research in this field.

In the past few decades, substantial evidence has been reported to assess the impact of PM_2.5_ on the likelihood of ASD during pregnancy and the prenatal period; researchers also provide related explanations including neuroinflammatory responses and oxidative stress (7, 39, 42, 43). Besides, early life exposure to PM_2.5_ is also considered a risk period related to ASD (16, 43, 44), as this is a time of particular vulnerability to external environmental factors, potentially influencing various aspects like cognition, brain structure, and the structural development of the heart, lungs, or atopy (10). However, studies specifically focusing on the association between PM_2.5_ and ASD symptoms are limited and their results vary considerably (12–14, 45). For instance, a Korean study involving children with ASD aged 5–14 found that high short-term exposure to PM_2.5_ was linked to a higher risk of hospital admissions, suggesting the detrimental impact of air pollution on ASD symptoms early in life (45). Similar findings were observed in a Chinese study that used the first three years after birth as the window period to examine the PM_2.5_-ASD odds association (14). In contrast, Tara et al. found no evidence of an association between ASD severity and PM_2.5_ exposure during pregnancy and the first year of life (13), and Santos et al. also reported that exposure to PM after the first year of life was not significantly associated with higher symptom severity (12). These conflicting conclusions might stem from differences in exposure levels and inadequate consideration of the true exposure experiences. Specially, we collected time of diagnosis, and identifying specific time intervals as exposure window periods. This approach allows us to address the temporality of associations commonly not be sufficient illustrated in previous studies. While we reported no relationship between total scores and symptoms, we identified detrimental effects of PM_2.5_ exposure occurring 13–18 months after birth, which could lead to an increased risk of more severe stereotypic behaviour symptoms. This aligns with published literature indicating that the second year of life is a critical window period related to increased ASD risks (15, 16). Our study also highlights the importance of this period, which coincides with critical periods of child brain development. As analysis from the largest neuroimaging dataset showed that the rate of growth (velocity) for total white matter volume (WMV) peaked in infancy and early childhood at around 2.4 years, and cortical thickness peaked distinctly early at 1.7 years, with the brain reaching approximately 80% of its maximum size by age 3 (46). Another explanation is that children are more likely to breathe more external air when they are outdoors during the second and third years after birth than in the first year (16).

Our study further investigated the relationship between greenness and ASD symptoms, a topic less explored in prior research. Our results reveal a negative correlation between increased greenness and the severity of ASD symptoms, affecting not only overall scores but also the subscales of relating and social independence. Notably, we observed a stronger link between greenness and symptoms during the second year of a child's life, followed by the first year. Previous studies have also reported the advantages of green spaces in reducing the risk of ASD in children. For instance, a study involving children aged 5–12 from 543 California public elementary schools found a decreased risk of ASD with higher green space exposure, including forests, grasslands, and tree canopies (27). Similarly, Pagalan et al.'s 2022 cohort study of 129,222 Canadian births reported lower ASD risks associated with increased prenatal greenness (28). Chen et al.'s 2023 analysis indicated that greenness in the first 1–3 years post-birth reduces ASD incidence risks (26). Unlike these studies, which focused on ASD risk, our research extended previous work by setting ASD symptoms as the dependent outcome. Nevertheless, our findings also confirmed the beneficial effects of greenness on ASD, as reported in previous studies. Our research emphasizes early-life exposure to assess the greenness-ASD link, a crucial period for brain growth and development in early childhood. While the biological mechanisms underlying the benefits of greenness on ASD remain unclear, several theories support our findings. Generally, green spaces offer environmental advantages by mitigating triggers like air and noise pollution and high temperatures (47). Interaction with natural settings and biodiversity enhances capabilities, specifically physical activities and social engagements (48, 49). For instance, it aids in developing communication skills, understanding and following simple instructions, and regulating daily behavior. These skills are crucial for the rehabilitation and symptom relief of children with autism. Natural environment also promotes psychological restoration, enhancing relating, attention, memory, and stress reduction, and may decrease sleep disturbances, further easing severe ASD symptoms (50). Also, natural vegetation can improve the human microbiome and its immunomodulatory capacity, linked to enriched skin and gut microbial diversity, in turn, may enhance brain development, possibly through immune response regulation (25, 51). Additionally, the associations between green space and childhood development were more pronounced among boys than girls (52), which further suggesting potential benefits for autistic children, the majority of whom are boys. Thus, our results emphasized the significance of developing green spaces, particularly in areas undergoing rapid urbanization.

This study presents a crucial advancement in understanding the interaction between PM_2.5_ and greenness on ASD symptoms, uncovering a synergistic effect where increased PM_2.5_ and reduced greenness correlate with heightened risks of severe ASD symptoms. In other words, our findings imply that increasing greenness while diminishing air pollution could potentially alleviate the progression of more severe symptoms in children with ASD. Previous research has underscored the mitigating influence of greenness on PM_2.5_ and birth outcomes (53, 54). Indeed, greenness confers a plethora of benefits, including shielding against air pollution and diminishing the detrimental effects of toxic substances in polluted air. A notable discovery is the protective role of early exposure to greenness (i.e., 6 months after birth) against the adverse effects of air pollution encountered 13–18 months after birth. This protective impact is significant both for overall ASD symptom scores and specific sub-scales, namely, language development. Literature supports the association of early childhood exposure to greenness with improved visual memory in mid-childhood (55), enhanced working memory, and decreased inattentiveness in 7-year-old children (31) and as well as better neurodevelopmental assessments in 2-year-olds (18). Researchers emphasize that the protective benefits of green spaces accumulate over time and highlight their continued importance throughout childhood (56). For instance, there exists a cumulative relationship between green space exposure and the development of the human microbiome, such as early childhood gut microbiota, which potentially strengthens the human immune system and lays the groundwork for a fundamentally healthy bodily environment (57, 58). Engagement with natural environments is regarded as a positive experience, fostering engagement, control, and stress-processing abilities; this interaction promotes psychological restoration, and positively influences ecological behaviors (59), hence, equipping individuals to better handle environmental challenges later in life. Furthermore, our sensitivity analyses indicated that co-exposure to PM_2.5_ and a lack of greenness was more closely associated with higher symptom severity than the combination of greenness and PM_10_. This is primarily due to the smaller size of PM_2.5_ particles, which are more likely to penetrate the lungs and cross the blood-brain barrier, potentially leading to greater neurodevelopmental issues among autistic children. Consequently, our findings advocate for the enhancement of accessible green spaces alongside improvements in air quality.

Our study acknowledges several limitations. Firstly, due to the unavailability of exact home addresses, we assumed that participants living in the same district were exposed to similar levels, potentially introducing measurement bias. This assumption was necessary as most caregivers, wary of the stigma associated with ASD, were reluctant to provide addresses during the process of interview. Addressing this limitation in future research is important. Secondly, it is unavoidable that the onset of early symptoms of ASD may not align with the time of diagnosis, which could affect the accuracy of our findings. Thirdly, no additional information was present potentially related to environmental exposure, such as time spent outdoors or frequency of using green spaces, which can significantly affect the impact of air pollution or greenness on ASD symptoms. Fourthly, our study focused on children with ASD recruited from urban special education schools, where there is a higher availability of ASD-diagnosed subjects. Future studies should extend to rural populations to enhance the generalizability of our findings. We acknowledge that the limited sample size may impact the generalization of our results. We also emphasize that our findings do not establish a causal link; they are merely correlational. Changes in ASD symptoms post-diagnosis could introduce some bias. However, as the ABC scale assesses both current and past behaviors, we are confident in the thorough documentation of symptoms. Lastly, we used PM_2.5_ as the primary air pollution exposure because it is the most widely discussed pollutant and has significantly contributed to the risks of ASD. In contrast, evidence levels regarding other air pollutants and their association with ASD or its severity is poor (3). Future research should focus on elucidating the biological mechanisms linking multiple air pollutants to ASD, and exploring the interaction effects with other environmental factors on ASD severity. Despite these limitations, our study addresses gaps in previous research by considering the temporality of associations and utilizing diagnosis timing for a more precise interpretation of the relationship between environmental factors and ASD symptoms. The ABC scale, suitable for a wide range of children's ages, offers multiple symptom assessments. We concurrently examined the impacts of air pollution and green spaces on ASD symptoms, conducted interaction and mediation analyses across different exposure periods, and provided new insights into the early-life benefits of green spaces. This enhances the existing body of research and deepens our understanding of these associations.

## 5 Conclusions

In conclusion, our research lends support to the hypothesis that severe symptoms of ASD are linked to exposure to PM_2.5_ and a scarcity of green spaces. Notably, our findings indicate that the second year of life is a particularly sensitive period in this context. We observed a synergistic effect in the combined influence of high PM_2.5_ levels and limited greenness, suggesting an additive interaction. This synergistic effect was also pronounced with high PM_2.5_ exposures during the 13–18 months after birth period, coupled with low exposure to green spaces during six months after birth, in relation to ASD symptoms. Our study did not find substantial evidence supporting a mediating role of greenness in the relationship between PM_2.5_ and ASD symptoms. This aspect of our research adds a good addition to current knowledge on the influences of environmental factors on ASD symptoms and also enhance our understanding of the potential dual benefits of reducing air pollution and increasing access to green spaces, particularly during the critical early years of children's lives who are diagnosed with ASD.

## Data Availability

The original contributions presented in the study are included in the article/Supplementary Material, further inquiries can be directed to the corresponding authors.
