# Supplementary material for: Independent and combined effects of fine particulate matter and greenness on autism spectrum disorder symptoms: investigating sensitive periods of exposure in the early two years of life
Source: Front Pediatr. 2025 Apr 10;13:1561476. doi: 10.3389/fped.2025.1561476 (PMC12018330; doi:10.3389/fped.2025.1561476)
Supplement: Supplementary file 1 [file Datasheet1.docx]

**Supplementary materials for** **Independent and Combined Effects of Fine Particulate Matter and Greenness on Autism Spectrum Disorder Symptoms: Investigating Sensitive Periods of Exposure in the Early Two Years of Life**

Yi Liu^1^, Wensu Zhou^2,3^, Meng Liu^1^, Yichao Wang^1^, Shu Chen^1*^, Xiyue Xiong^1*^

**
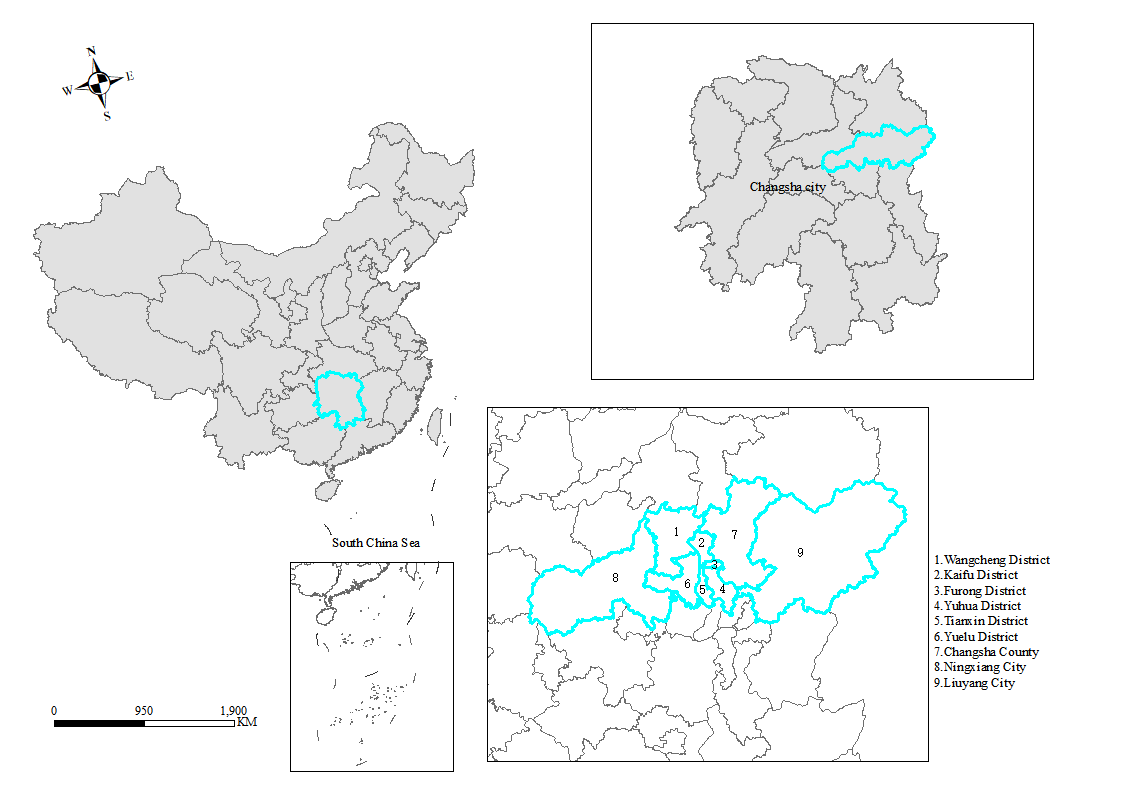
**

**Figure S1 Map of Changsha city included in the study**

**Table S1** **Associations between PM_2.5_ and greenness exposure and symptoms by considering the period before diagnosis as a sensitive window of exposure**

| Exposure window period | N | Continuous dependent variables β(95%CI) | |  | Categorical dependent variables  OR(95%CI) | |
| --- | --- | --- | --- | --- | --- | --- |
|  |  | PM_2.5_ | Greenness |  | PM_2.5_ | Greenness |
| Dependent variable: ABC total scores |  |  |  |  |  |  |
| First year before diagnosis | 108 | 0.01 (-0.13,0.15) | -0.03 (-0.12,0.05) |  | 1.03 (0.38,2.79) | 1.09 (0.63,1.90) |
| Second year before diagnosis | 89 | -0.04 (-0.17,0.09) | -0.09 (-0.18,0.01) |  | 0.87 (0.32,2.34) | 0.61 (0.28,1.32) |
| Dependent variable: ABC sensory |  |  |  |  |  |  |
| First year before diagnosis | 108 | -0.03 (-0.18,0.11) | 0.04 (-0.05,0.12) |  | 0.70 (0.27,1.80) | 1.39 (0.80,2.41) |
| Second year before diagnosis | 89 | -0.03 (-0.17,0.11) | 0.00 (-0.10,0.10) |  | 0.76 (0.32,1.82) | 1.28 (0.65,2.50) |
| Dependent variable: ABC relating |  |  |  |  |  |  |
| First year before diagnosis | 108 | -0.07 (-0.19,0.04) | -0.04 (-0.10,0.03) |  | 0.66 (0.24,1.81) | 0.80 (0.46,1.37) |
| Second year before diagnosis | 89 | -0.05 (-0.16,0.06) | -0.06 (-0.14,0.02) |  | 1.03 (0.44,2.42) | 0.76 (0.40,1.44) |
| Dependent variable: ABC stereotypic behaviour |  |  |  |  |  |  |
| First year before diagnosis | 108 | 0.06 (-0.11,0.23) | -0.06 (-0.16,0.04) |  | 1.60 (0.06,4.25) | 0.72 (0.41,1.28) |
| Second year before diagnosis | 89 | -0.11 (-0.26,0.04) | -0.08 (-0.19,0.03) |  | 0.46 (0.17,1.22) | **0.46 (0.22,0.97)** |
| Dependent variable: ABC language |  |  |  |  |  |  |
| First year before diagnosis | 108 | 0.02 (-0.12,0.16) | 0.03 (-0.05,0.12) |  | 1.19 (0.45,3.16) | 1.11 (0.63,1.97) |
| Second year before diagnosis | 89 | -0.03 (-0.17,0.11) | 0.00 (-0.10,0.10) |  | 1.53 (0.61,3.84) | 0.74 (0.38,1.44) |
| Dependent variable: ABC social independence |  |  |  |  |  |  |
| First year before diagnosis | 108 | 0.00 (-0.09,0.09) | -0.04 (-0.10,0.01) |  | 0.89 (0.34,2.38) | 0.67 (0.37,1.19) |
| Second year before diagnosis | 89 | -0.04 (-0.13,0.04) | -0.06 (-0.12,0.00) |  | 0.91 (0.36,2.31) | 0.55 (0.27,1.09) |

***Note:***

Characters in bold means statistical significance at p = 0.05. Models were adjusted for gender of children, race/ethnicity, parity, maternal age at delivery, age of male parents, maternal marital state, maternal occupation, maternal education level, year of birth, and season of year.

**Table S2 Dose-response associations between PM_2.5_ and greenness in relation to ASD symptoms**

| Measurement | Exposure window period | N | Continuous dependent variables | |  | Categorical dependent variables | |
| --- | --- | --- | --- | --- | --- | --- | --- |
|  |  |  | Non-liner P values for PM_2.5_ | Non-liner P values for greenness |  | Non-liner P values for PM_2.5_ | Non-liner P values for greenness |
| ABC total scores | Stage 1 | 108 | 0.60 | 0.87 |  | 0.73 | 0.30 |
|  | Stage 2 | 108 | 0.94 | 0.85 |  | 0.33 | 0.45 |
|  | Stage 3 | 103 | 0.49 | 0.43 |  | 0.79 | 0.92 |
|  | Stage 4 | 89 | 0.12 | 0.50 |  | 0.08 | 0.05 |
|  | Stage 5 | 108 | 0.79 | 0.34 |  | 0.55 | 0.50 |
|  | Stage 6 | 108 | 0.51 | 0.43 |  | 0.78 | 0.36 |
| Sensory | Stage 1 | 108 | 0.68 | 0.51 |  | 0.71 | 0.35 |
|  | Stage 2 | 108 | 0.95 | 0.96 |  | 0.84 | 0.66 |
|  | Stage 3 | 103 | 0.31 | 0.52 |  | 0.58 | 0.13 |
|  | Stage 4 | 89 | 0.23 | 0.78 |  | 0.33 | 0.91 |
|  | Stage 5 | 108 | 0.26 | 0.83 |  | 0.55 | 0.72 |
|  | Stage 6 | 108 | 0.98 | 0.51 |  | 0.91 | 0.23 |
| Relating | Stage 1 | 108 | 0.61 | 0.92 |  | 0.65 | 0.16 |
|  | Stage 2 | 108 | 0.99 | 0.85 |  | 0.74 | 0.33 |
|  | Stage 3 | 103 | 0.12 | 0.53 |  | 0.39 | 0.95 |
|  | Stage 4 | 89 | 0.44 | 0.64 |  | 0.58 | 0.63 |
|  | Stage 5 | 108 | 0.72 | 0.35 |  | 0.62 | 0.61 |
|  | Stage 6 | 108 | 0.62 | 0.52 |  | 0.79 | 0.54 |
| Stereotypic behaviour | Stage 1 | 108 | 0.86 | 0.97 |  | 0.82 | 0.98 |
|  | Stage 2 | 108 | 0.27 | 0.93 |  | 0.21 | 0.55 |
|  | Stage 3 | 103 | 0.06 | 0.60 |  | 0.10 | 0.24 |
|  | Stage 4 | 89 | 0.06 | 0.71 |  | **0.04** | 0.30 |
|  | Stage 5 | 108 | 0.82 | 0.64 |  | 0.93 | 0.66 |
|  | Stage 6 | 108 | 0.91 | 0.65 |  | 0.50 | 0.82 |
| Language | Stage 1 | 108 | 0.28 | 0.72 |  | 0.24 | 0.31 |
|  | Stage 2 | 108 | 0.51 | 0.98 |  | 0.47 | 0.55 |
|  | Stage 3 | 103 | 0.82 | 0.20 |  | 0.82 | 0.17 |
|  | Stage 4 | 89 | 0.45 | 0.51 |  | 0.09 | 0.19 |
|  | Stage 5 | 108 | 0.44 | 0.84 |  | 0.82 | 0.98 |
|  | Stage 6 | 108 | 0.51 | 0.77 |  | 0.30 | 0.94 |
| Social independence | Stage 1 | 108 | 0.43 | **0.01** |  | 0.46 | **0.03** |
|  | Stage 2 | 108 | 0.43 | 0.53 |  | 0.63 | 0.80 |
|  | Stage 3 | 103 | 0.78 | 0.17 |  | 0.91 | 0.19 |
|  | Stage 4 | 89 | 0.06 | 0.31 |  | 0.13 | 0.79 |
|  | Stage 5 | 108 | 0.39 | 0.98 |  | 0.49 | 0.81 |
|  | Stage 6 | 108 | 0.69 | 0.87 |  | 0.51 | 0.66 |

***Note:***

Characters in bold means statistical significance at p = 0.05. Models were adjusted for gender of children, race/ethnicity, parity, maternal age at delivery, age of male parents, maternal marital state, maternal occupation, maternal education level, year of birth, and season of year. 6 months after date of birth (Stage 1), 7 months after date of birth - 12 months after date of birth (stage 2), 13 months after date of birth - 18 months after date of birth (stage 3), 19 months after date of birth - 24 months after date of birth (stage 4), first year after birth (i.e., age 1) (stage 5), second year after birth (i.e., age 2) (stage 6).

**Table S3 Additive interaction between PM_2.5_ and greenness on overall ASD symptoms**

| Exposure window period for PM_2.5_ | Exposure window period for NDVI | N | ABC | | |
| --- | --- | --- | --- | --- | --- |
|  |  |  | RERI | 95%CI | |
| Stage 1 | Stage 1 | 108 | **0.92** | **0.27** | **1.57** |
|  | Stage 2 | 108 | 0.45 | -0.74 | 1.64 |
|  | Stage 3 | 103 | 0.90 | -0.18 | 1.99 |
|  | Stage 4 | 89 | -0.20 | -2.47 | 2.08 |
| Stage 2 | Stage 1 | 108 | 0.25 | -1.05 | 1.55 |
|  | Stage 2 | 108 | -2.29 | -7.16 | 2.57 |
|  | Stage 3 | 103 | -0.05 | -2.10 | 2.00 |
|  | Stage 4 | 89 | -3.97 | -13.10 | 5.12 |
| Stage 3 | Stage 1 | 103 | **1.16** | **0.54** | **1.77** |
|  | Stage 2 | 103 | 0.75 | -0.59 | 2.08 |
|  | Stage 3 | 103 | **1.01** | **0.26** | **1.77** |
|  | Stage 4 | 89 | 0.56 | -1.05 | 2.17 |
| Stage 4 | Stage 1 | 89 | -7.70 | -25.30 | 9.88 |
|  | Stage 2 | 89 | -21.40 | -69.10 | 26.40 |
|  | Stage 3 | 89 | -22.00 | -74.50 | 30.40 |
|  | Stage 4 | 89 | -6.41 | -20.40 | 7.59 |
| Stage 5 | Stage 5 | 108 | -0.64 | -3.93 | 2.66 |
|  | Stage 6 | 108 | -2.51 | -8.78 | 3.76 |
| Stage 6 | Stage 6 | 108 | -2.06 | -6.03 | 1.92 |

***Note:***

Characters in bold means statistical significance at p = 0.05. PM_2.5_ / NDVI was divided into two categories: <Median and ≥ median; ABC was divided into two categories: <68 and ≥ 68; RERI relative excess risk due to interaction. Models were adjusted for gender of children, race/ethnicity, parity, maternal age at delivery, age of male parents, maternal marital state, maternal occupation, maternal education level, year of birth, and season of year. 6 months after date of birth (Stage 1), 7 months after date of birth - 12 months after date of birth (stage 2), 13 months after date of birth - 18 months after date of birth (stage 3), 19 months after date of birth - 24 months after date of birth (stage 4), first year after birth (i.e., age 1) (stage 5), second year after birth (i.e., age 2) (stage 6).

**Table S4 Additive interaction between PM_2.5_ and greenness on five sub-scales of symptoms**

| Exposure window period for PM_2.5_ | Exposure window period for NDVI | Sensory |  | Relating |  | Stereotypic behaviour |  | Language |  | Social independence |
| --- | --- | --- | --- | --- | --- | --- | --- | --- | --- | --- |
|  |  | RERI (95%CI) |  | RERI (95%CI) |  | RERI (95%CI) |  | RERI (95%CI) |  | RERI (95%CI) |
| Stage 1 | Stage 1 | **0.76 (0.01,1.51)** |  | 0.58 (-0.77,1.93) |  | 2.82 (-0.77,6.40) |  | 0.86 (-1.39,3.11) |  | 0.56 (-0.84,1.97) |
|  | Stage 2 | 0.37 (-0.53,1.27) |  | 0.79 (-1.57,3.15) |  | 1.62 (-4.29,7.54) |  | 0.81 (-1.58,3.21) |  | -2.43 (-9.39,4.53) |
|  | Stage 3 | 0.69 (-0.15,1.53) |  | 0.29 (-1.94,2.51) |  | 4.77 (-3.01,12.55) |  | 1.09 (-0.97,3.15) |  | -2.43 (-9.39,4.53) |
|  | Stage 4 | 0.54 (-0.37,1.44) |  | 1.67 (-2.92,6.26) |  | 9.26 (-16.76,35.27) |  | 0.24 (-1.52,2.01) |  | 0.62 (-0.62,1.86) |
| Stage 2 | Stage 1 | 0.08 (-1.26,1.42) |  | 0.24 (-0.91,1.40) |  | -0.34 (-2.19,1.51) |  | 0.45 (-1.54,2.45) |  | -3.37 (-11.89,5.14) |
|  | Stage 2 | 0.37 (-1.00,1.73) |  | -1.11 (-4.69,2.46) |  | -0.59 (-3.20,2.01) |  | -0.04 (-1.92,1.84) |  | -0.07 (-3.54,3.40) |
|  | Stage 3 | 0.39 (-0.72,1.49) |  | -0.01 (-1.70,1.68) |  | 0.17 (-1.36,1.70) |  | -2.37 (-7.15,2.41) |  | -15.04 (-45.7,15.62) |
|  | Stage 4 | -0.23 (-2.83,2.37) |  | -9.92 (-29.54,9.70) |  | -0.38 (-2.93,2.18) |  | -0.24 (-2.49,2.01) |  | -0.77 (-4.16,2.63) |
| Stage 3 | Stage 1 | -0.03 (-1.77,1.70) |  | 1.2 (-0.18,2.59) |  | 3.81 (-0.81,8.42) |  | **1.4 (0.09,2.72)** |  | -0.32 (-3.19,2.55) |
|  | Stage 2 | -2.38 (-6.85,2.09) |  | 1.96 (-2.84,6.75) |  | 5.32 (-7.11,17.75) |  | -1.03 (-4.36,2.29) |  | 1.39 (-3.44,6.23) |
|  | Stage 3 | 0.36 (-0.92,1.63) |  | 0.69 (-1.00,2.39) |  | 3.14 (-0.71,6.99) |  | 1.16 (-0.08,2.40) |  | 0.29 (-1.15,1.73) |
|  | Stage 4 | -0.74 (-3.67,2.19) |  | 1.49 (-3.01,5.99) |  | 4.07 (-6.57,14.72) |  | 0.23 (-1.53,1.99) |  | -0.68 (-4.59,3.23) |
| Stage 4 | Stage 1 | 8.15 (-15.94,32.24) |  | -3.53 (-11.25,4.19) |  | -0.91 (-6.3,4.48) |  | -1.71 (-6.11,2.69) |  | -5.05 (-18.92,8.82) |
|  | Stage 2 | -3.32 (-17.75,11.10) |  | -12.76 (-36.42,10.9) |  | -1.57 (-6.67,3.52) |  | -5.76 (-20.59,9.06) |  | -8.49 (-30.88,13.9) |
|  | Stage 3 | 5.65 (-12.61,23.91) |  | -3.27 (-11.2,4.66) |  | -0.32 (-4.79,4.14) |  | -3.02 (-9.42,3.38) |  | -2.63 (-10.46,5.21) |
|  | Stage 4 | 0.22 (-8.21,8.64) |  | -16.44 (-47.69,14.81) |  | 0.61 (-1.4,2.61) |  | -3.19 (-10.86,4.48) |  | -1.52 (-10.63,7.59) |
| Stage 5 | Stage 5 | 0.15 (-1.40,1.70) |  | -0.74 (-4.06,2.58) |  | 2.11 (-8.6,12.81) |  | 0.7 (-0.03,1.43) |  | -2.65 (-11.43,6.12) |
|  | Stage 6 | 0.19 (-1.11,1.50) |  | -0.95 (-4.39,2.50) |  | 0.31 (-20.5,21.1) |  | 0.43 (-0.54,1.39) |  | -2.44 (-10.92,6.04) |
| Stage 6 | Stage 6 | -1.95 (-8.59,4.69) |  | -1.36 (-4.67,1.95) |  | -1.59 (-15.97,12.8) |  | 0.09 (-1.19,1.37) |  | -0.33 (-3.59,2.93) |

***Note:***

Characters in bold means statistical significance at p = 0.05. PM_2.5_ / NDVI was divided into two categories: <Median and ≥ median; Scores for each sub-scale was divided into two categories: <68 and ≥ 68; RERI relative excess risk due to interaction. Models were adjusted for gender of children, race/ethnicity, parity, maternal age at delivery, age of male parents, maternal marital state, maternal occupation, maternal education level, year of birth, and season of year. 6 months after date of birth (Stage 1), 7 months after date of birth - 12 months after date of birth (stage 2), 13 months after date of birth - 18 months after date of birth (stage 3), 19 months after date of birth - 24 months after date of birth (stage 4), first year after birth (i.e., age 1) (stage 5), second year after birth (i.e., age 2) (stage 6).


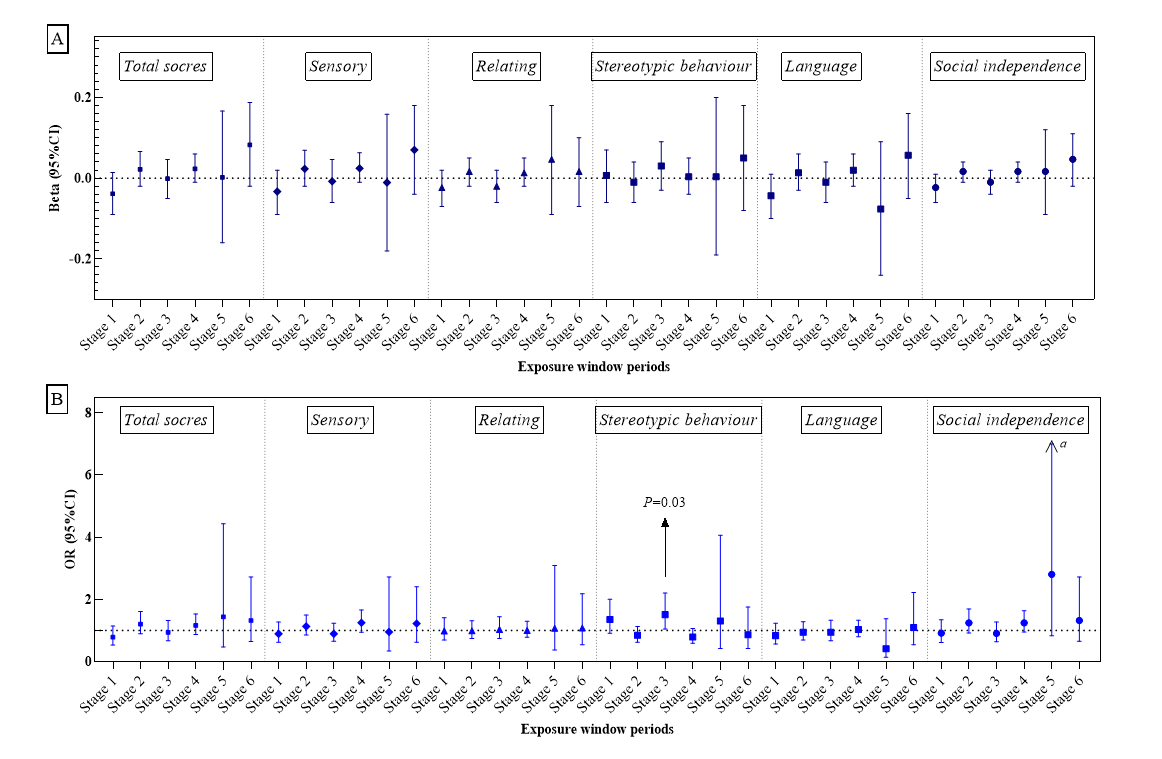


**Figure S2 Associations between PM_10_ exposure and ASD symptoms.** (A): Each 10 ug/m3 increase in PM_10_ and symptoms defined as continues variables; (B) Each 10 ug/m3 increase in PM_10_ and symptoms defined as categorical variables (<68 vs. ≥ 68); Sample size for stage 1 to 6 were 108, 108, 103, 89, 108, and 108, respectively. Models were adjusted for gender of children, race/ethnicity, parity, maternal age at delivery, age of male parents, maternal marital state, maternal occupation, maternal education level, year of birth, and season of year. a = 9.48. 6 months after date of birth (Stage 1), 7 months after date of birth - 12 months after date of birth (stage 2), 13 months after date of birth - 18 months after date of birth (stage 3), 19 months after date of birth - 24 months after date of birth (stage 4), first year after birth (i.e., age 1) (stage 5), second year after birth (i.e., age 2) (stage 6).


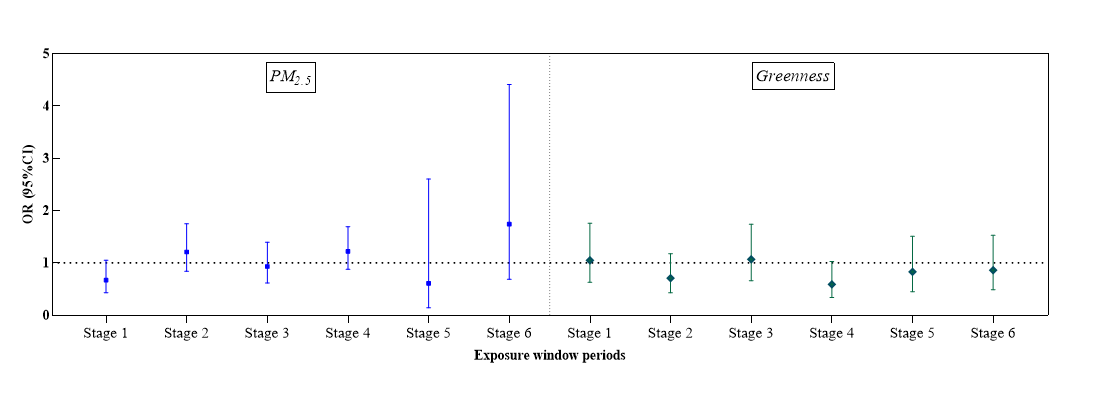


**Figure S3 Associations between PM_2.5_ and greenness and ASD symptoms (<53 vs. ≥ 53).** Sample size for stage 1 to 6 were 108, 108, 103, 89, 108, and 108, respectively. Models were adjusted for gender of children, race/ethnicity, parity, maternal age at delivery, age of male parents, maternal marital state, maternal occupation, maternal education level, year of birth, and season of year. 6 months after date of birth (Stage 1), 7 months after date of birth - 12 months after date of birth (stage 2), 13 months after date of birth - 18 months after date of birth (stage 3), 19 months after date of birth - 24 months after date of birth (stage 4), first year after birth (i.e., age 1) (stage 5), second year after birth (i.e., age 2) (stage 6).

**Table S5 Additive interaction between PM and greenness exposure on ASD symptoms based on logistic regression models**

| Exposure window period for PM | Exposure window period for NDVI | N | Estimate (95%CI) for additive effect of PM_10_ and greenness on scores | | |  | Estimate (95%CI) for additive effect of PM_2.5_ and greenness on ABC total scores (<53 vs. ≥ 53) | | |
| --- | --- | --- | --- | --- | --- | --- | --- | --- | --- |
| Stage 3 | Stage 1 | 103 |  |  |  |  |  |  |  |
| Low - PM (<P_50_) | High - NDVI (≥P_50_) |  | 1[Ref] |  |  |  | 1[Ref] |  |  |
| Low - PM (<P_50_) | Low - NDVI (<P_50_) |  | 0.41 | 0.08 | 1.97 |  | 0.16 | 0.03 | 0.83 |
| High - PM (≥P_50_) | High - NDVI (≥P_50_) |  | 0.48 | 0.10 | 2.18 |  | 0.35 | 0.07 | 1.73 |
| High - PM (≥P_50_) | Low - NDVI (<P_50_) |  | 0.55 | 0.16 | 1.77 |  | 0.86 | 0.24 | 3.07 |
| RERI for total scores (<68 vs. ≥ 68) | |  | 0.66 | -0.32 | 1.63 |  | **1.35** | **0.38** | **2.31** |
| Stage 3 | Stage 3 | 103 |  |  |  |  |  |  |  |
| Low - PM (<P_50_) | High - NDVI (≥P_50_) |  | 1[Ref] |  |  |  | 1[Ref] |  |  |
| Low - PM (<P_50_) | Low - NDVI (<P_50_) |  | 1.13 | 0.26 | 4.95 |  | 0.19 | 0.03 | 0.96 |
| High - PM (≥P_50_) | High - NDVI (≥P_50_) |  | 1.92 | 0.52 | 7.46 |  | 0.27 | 0.06 | 1.18 |
| High - PM (≥P_50_) | Low - NDVI (<P_50_) |  | 0.66 | 0.15 | 2.80 |  | 0.68 | 0.19 | 2.40 |
| RERI for total scores (<68 vs. ≥ 68) | |  | -1.40 | -4.68 | 1.88 |  | **1.22** | **0.48** | **1.96** |
| Stage 3 | Stage 1 | 103 |  |  |  |  |  |  |  |
| Low - PM (<P_50_) | High - NDVI (≥P_50_) |  | 1[Ref] |  |  |  | 1[Ref] | - | - |
| Low - PM (<P_50_) | Low - NDVI (<P_50_) |  | 0.35 | 0.07 | 1.75 |  | - | - | - |
| High - PM (≥P_50_) | High - NDVI (≥P_50_) |  | 0.53 | 0.09 | 2.80 |  | - | - | - |
| High - PM (≥P_50_) | Low - NDVI (<P_50_) |  | 1.08 | 0.32 | 3.64 |  | - | - | - |
| RERI for scores of language (<16.5 and ≥16.5) | |  | 1.20 | -0.08 | 2.48 |  | - | - | - |

***Note:***

Characters in bold means statistical significance at p = 0.05. PM_2.5_ / NDVI was divided into two categories: <Median and ≥ Median; Total ABC scores were divided into two categories as a binary variable; RERI relative excess risk due to interaction. Models were adjusted for gender of children, race/ethnicity, parity, maternal age at delivery, age of male parents, maternal marital state, maternal occupation, maternal education level, year of birth, and season of year. 6 months after date of birth (Stage 1), 7 months after date of birth - 12 months after date of birth (stage 2), 13 months after date of birth - 18 months after date of birth (stage 3), 19 months after date of birth - 24 months after date of birth (stage 4), first year after birth (i.e., age 1) (stage 5), second year after birth (i.e., age 2) (stage 6).
